# Efficacy of a Web-Based Self-Management Enhancing Program for Patients with Rheumatoid Arthritis: Explorative Randomized Controlled Trial

**DOI:** 10.2196/12463

**Published:** 2019-04-30

**Authors:** Rixt Zuidema, Sandra van Dulmen, Maria Nijhuis-van der Sanden, Inger Meek, Cornelia van den Ende, Jaap Fransen, Betsie van Gaal

**Affiliations:** 1 Scientific Institute for Quality of Healthcare Radboud Institute for Health Sciences Radboud University Medical Center Nijmegen Netherlands; 2 Department of Primary and Community Care Radboud Institute for Health Sciences Radboud University Medical Center Nijmegen Netherlands; 3 Netherlands Institute for Health Services Research Utrecht Netherlands; 4 Faculty of Health and Social Sciences University of South-Eastern Norway Drammen Norway; 5 Department of Rheumatology Radboud Institute for Health Sciences Radboud University Medical Center Nijmegen Netherlands; 6 Department of Rheumatology and Pharmacy Sint Maartenskliniek Nijmegen Netherlands; 7 Institute of Nursing Hogeschool van Arnhem en Nijmegen University of Applied Sciences Nijmegen Netherlands

**Keywords:** self-management, internet, arthritis, rheumatoid

## Abstract

**Background:**

Web-based self-management enhancing programs have the potential to support patients with rheumatoid arthritis (RA) in their self-management; for example, improve their health status by increasing their self-efficacy or taking their prescribed medication. We developed a Web-based self-management enhancing program in collaboration with RA patients and professionals as co-designers on the basis of the intervention mapping framework. Although self-management programs are complex interventions, it is informative to perform an explorative randomized controlled trial (RCT) before embarking on a larger trial.

**Objective:**

This study aimed to evaluate the efficacy of a Web-based self-management enhancing program for patients with RA and identify outcome measures most likely to capture potential benefits.

**Methods:**

A multicenter exploratory RCT was performed with an intervention group and a control group. Both groups received care as usual. In addition, the intervention group received 12 months of access to a Web-based self-management program. Assessment occurred at baseline, 6 months, and 12 months. Outcome measures included self-management behavior (Patient Activation Measurement, Self-Management Ability Scale), self-efficacy (Rheumatoid Arthritis task-specific Self-Efficacy, Perceived Efficacy in Patient-Physician Interaction), general health status (RAND-36), focus on fatigue (Modified Pain Coping Inventory for Fatigue), and perceived pain and fatigue (Numeric Rating Scales). A linear mixed model for repeated measures, using the intention-to-treat principle, was applied to study differences between the patients in the intervention (n=78) and control (n=79) groups. A sensitivity analysis was performed in the intervention group to study the influence of patients with high (N=30) and low (N=40) use of the intervention.

**Results:**

No positive effects were found regarding the outcome measurements. Effect sizes were low.

**Conclusions:**

Based on these results, it is not possible to conclude on the positive effects of the intervention or to select outcome measures to be regarded as the primary/main or secondary outcomes for a future trial. A process evaluation should be performed to provide more insight into the low compliance with and effectiveness of the intervention. This can determine for whom this sort of program will work and help to fine-tune the inclusion criteria.

**Trial Registration:**

Netherlands Trial Register NTR4871; https://www.trialregister.nl/trial/4726

## Introduction

### Background

Rheumatoid arthritis (RA) is one of the most prevalent chronic conditions, with a pervasive impact on daily life [1]. Despite the introduction of biological therapies and conventional disease-modifying antirheumatic drugs, RA patients experience a high level of pain [2] and fatigue [3,4], which leads to disabilities like restrictions in work participation [5,6] and leisure activities [7-9]. Moreover, many RA patients experience disease-related psychological problems, like depressive mood and helplessness [10,11].

To optimally manage the consequences of RA and reduce the impact of the disease on patients in daily life, effective self-management programs are needed. Web-based self-management programs can easily reach a large group of RA patients in their own place and time and provide more anonymity than face-to-face programs. Studies have shown that patients feel more comfortable sharing sensitive information like reports on daily activity or feelings online [12]. Other advantages are the possibility of tailoring information, avoiding waiting lists, and 24-hour availability [13].

Studies about Web-based self-management programs have shown to be effective in RA patients on several health outcomes, including increased self-efficacy, knowledge and physical activity [14], less pain, disability and depression, and reduction in the overuse of medication and the number of visits to physicians [15-17]. However, many of the programs are developed without end-user involvement. Consequently, these programs may not suit patient support needs for self-management as patient preferences for program use are not well known [18,19].

To guarantee optimal patient involvement, we developed a Web-based self-management program on the basis of intervention mapping (IM), called Reuma zelf te lijf (Coping with RA) [20-22]. According to the Medical Research Council (MRC), complex interventions such as this program can be evaluated in a randomized controlled trial (RCT); however, it is advised to first perform an explorative study investigating potential outcome measurements to be used in a larger trial [23].

### Objectives

Therefore, the present explorative RCT study in patients with RA was aimed as follows: (1) to explore the potential efficacy of a Web-based self-management enhancing program versus “usual care” on self-management behavior, self-efficacy, general health status, coping with fatigue and the level of pain and fatigue and to determine the effect sizes at 6 and 12 months after baseline, and (2) to identify outcome measures most likely to capture the potential benefits covered by the performance objectives, by exploring their floor and ceiling effects at baseline.

## Methods

### Design

A multicenter exploratory RCT was conducted in 2 Dutch hospitals, The Radboudumc (a University hospital) and the Sint Maartenskliniek (a specialized hospital in rheumatology, rehabilitation, and orthopedic surgery), both located in Nijmegen, the Netherlands. An intervention and a control group were compared at 6 and 12 months after baseline on 6 outcome measurements to explore the efficacy of the Web-based program and to identify outcome measures [20]. The trial is registered at the Netherlands Trial Register (ID: NTR4871).

### Ethical Approval

The medical ethics committee of Arnhem-Nijmegen approved this study (No. 2014-1208).

### Participants

Between December 2014 and June 2015, patients with a diagnosis of RA aged 18 years or older were invited by a letter to participate in this study, in collaboration with rheumatologists, until the required number of 190 patients was reached. Patients received the following: (1) information about the study, (2) a questionnaire for screening eligibility, and (3) an informed consent form. Eligibility criteria were the ability to speak and read Dutch and having access to a computer with an internet connection. Patients receiving psychiatric or psychological treatment were excluded. Patients willing to participate were asked to return the informed consent with the completed questionnaire. When patients agreed to participate and were eligible, the researcher sent the patient an email with the baseline questionnaire.

### Randomization

Eligible patients were stratified by the hospital and randomly assigned to the intervention or control group by an independent statistician using an automated randomization program. The researcher informed the patients by post if they were allocated to the control or intervention group. Patients in the control group continued with their care as usual, which comprised medical treatment at the outpatient clinic. The patients in the intervention group received, in additional to their care as usual, 12 months of access to the intervention directly after randomization.

**Table 1:** Overview of the 9 modules and their performance objectives.

Module name	Performance objective: Patients need to...
Balancing activity and rest	...find balance between rest and activity; make choices when participating in daily life activities to keep balance
Setting boundaries	...set boundaries for their partner, relatives, colleagues and social environment
Asking for help and social support	...ask for social support or practical help from their partner, relatives, colleagues and social environment in daily life; ask for social support and practical help from colleagues; accept receiving social support or practical help from their partner, relatives, colleagues and social environment in daily life
Use of medicines	...take prescribed medication
Communication with health professionals	...prepare for a visit to a health professional; ask questions and/or express concerns during an appointment with a health professional
Use of assistive devices	...use, if necessary, assistive devices
Performing physical exercises	...perform daily physical exercises
Coping with worries	...cope with worries about RA^a^
Coping with RA	...cope with RA

^a^RA: rheumatoid arthritis.

### Intervention

#### Web-Based Self-Management Enhancing Program

The intervention was developed between January 2013 and July 2014 in collaboration with RA patients and professionals as co-designers [20,24]. The theory of planned behavior was used as the underlying theory and essential behavioral change techniques were applied to induce behavioral change formulated as performance objectives, selected according to the IM steps [21,25,26]. The Web-based self-management enhancing program comprises 9 modules with 13 performance objectives (Table 1) and a diary to track patients’ fatigue and pain over time [20]. Each module comprises 2-5 sessions, with informational and persuasive texts, videos with instructions and role models, exercises, and assignments. The program is unguided, and patients need to choose a module by their own and can work through it at their own pace whenever they want.

#### Implementation of the Web-Based Self-Management Enhancing Program

To implement the Web-based program and to increase use of the program by patients, 3 implementation strategies were deployed during the study: (1) patients received a written instruction manual for the program, (2) reminders to (re)visit the program were sent twice weekly via email, and (3) nurses brought the program to the attention of the intervention group participants during their consultation.

#### Measurements and Outcomes

All included patients who filled in the baseline questionnaire between January 2015 and June 2015 received a questionnaire after 6 months (T1) and 12 months (T2). At baseline, demographic and disease characteristics were assessed. Patient-reported outcome measurements were assessed at baseline and during follow-ups (T1 and T2). When patients preferred a paper questionnaire, a version was sent by post. When patients did not return the questionnaire at T1 but filled in the questionnaire at T2, this was indicated as a missing value at T1. Patients who did not return the T2 questionnaire are indicated as dropouts.

#### Baseline Characteristics

The following demographic and disease characteristics were assessed: age, gender, education level, employment status, disease duration, Numeric Rating Scales (NRS) pain/fatigue, Modified Health Assessment Questionnaire (M-HAQ) physical disability, and satisfaction with health status. The M-HAQ comprises 8 questions on difficulties in daily activities in the following domains: dressing, rising, eating, walking, hygiene, reaching, gripping, and usual activities. Patients responded on a 4-point scale, with a higher score indicating more difficulty in performing daily activities. Health satisfaction was assessed using 1 question about patients’ (dis)satisfaction about the course of their disease last week, with 4 response options, with a higher score meaning less satisfied than before and an *“I don't know”* option [27].

#### Outcome Measurements

Based on the theory of planned behavior, 6 outcome measures were relevant: self-management behavior, self-efficacy, general health status, coping with fatigue, and the level of pain and fatigue.

#### Self-Management Behavior

The Patient Activation Measurement (PAM-13) includes statements about individuals’ knowledge, confidence, and skills for self-management of their chronic illness behavior and the level of activation. It includes 13 items on a 5-point scale with a higher score indicating a higher level of patient activation. The scores of the 13 items are summarized as a total score. Total PAM scores were computed if at least 10 items were completed [28].

The short Self-Management Ability Scale (SMAS-S) comprises 18 items scored on a 6-point scale with a higher score indicating better self-management behavior [29].

#### Self-Efficacy

The Rheumatoid Arthritis task-specific Self-Efficacy (RASE) questionnaire comprises 28 items scored on a 5-point Likert scale. Higher scores reflect higher self-efficacy [30]. This questionnaire was translated into Dutch via forward-backward translation and decisions were based on consensus with a group of 5 researchers, 4 RA patients, and 1 RA patient who was a native English speaker. The Perceived Efficacy in Patient-Physician Interaction (PEPPI-5) comprises a 5-point Likert scale. A higher score reflects more confidence in patient interactions with their physician [31].

#### General Health Status

The RAND-36 comprises 36 questions measuring 8 dimensions: physical functioning, social functioning, physical role limitations, emotional role limitations, mental health, vitality, pain, perceived health-related quality of life and behavioral change, with various response options based on 3- to 6-point Likert scales, with a higher score indicating better perceived health-related quality of life. Scores were transformed to a 0-100-point scale for each subscale [32].

#### Level of Pain and Fatigue

Pain and fatigue were measured with NRS, ranging from 0 to 10 with 0 indicating no pain/fatigue and 10 indicating severe pain/very tired. For both outcomes, 2 questions were asked: the level of pain/fatigue today and the mean level of pain/fatigue during the last 2 weeks.

#### Coping with Fatigue

The Modified Pain Coping Inventory for Fatigue (MPCI-F) was used. This questionnaire is based on a subscale of the Pain Coping Inventory questionnaire and modified to assess coping with fatigue instead of coping with pain [33]. The questionnaire comprises 8 items to assess the focus on fatigue. A higher score reflects more focus on fatigue.

#### Statistical Analysis

Descriptive statistics were used to describe the control and intervention groups at baseline. *t* tests and Chi-square tests were used to analyze baseline differences. It was analyzed whether the patients who dropped out differed from the group that returned the questionnaire at T2 [34]. Between-group differences in outcomes were analyzed using a linear mixed model to account for repeated measurements and to handle missing data under the missing-at-random assumption. Differences between the intervention and control groups were analyzed at baseline, after 6 months (T1), and 12 months (T2). The fixed variables in the model were as follows: group (intervention/control), hospital (hospital 1 or hospital 2), age, gender, disease duration, education level, employment status, physical functioning (M-HAQ), and the interaction terms between measurement time points and groups. The first analysis was done using the intention-to-treat principle. Subsequently, a sensitivity analysis was performed to explore the influence of program use within the intervention group. The intervention group was divided into 3 groups: (1) a group with low usage (0-1 visits), (2) a group with moderate usage (2-5 visits), and (3) a group with high usage (6 or more visits). In the analysis, the group with moderate usage was left out to increase the contrast between the groups with low and high usage. *t* tests and Chi-square tests were performed to analyze between-group differences in demographics, disease-related characteristics, and outcomes at baseline, T1, and T2. Statistical significance was defined as *P*<.05.

For all outcome measurements, Cohen *d* was used to quantify effect sizes by calculating the difference in means, divided by the pooled within-group standard deviation [35]. Following Cohen definition of effect sizes, less than 0.4 was defined as a small effect, between 0.5 and 0.7 as moderate, and ≥0.8 was considered as a large effect [36]. Floor and ceiling effects were explored for all outcome measures by examining the percentage of minimum and maximum scores, which reflects the extent that patients scored the lowest or the highest score. For a 3- or 5-point Likert scale, floor and ceiling effects were defined as more than 80% of the patients scoring the lowest/highest. Statistical analyses were performed using SPSS version 22 (SPSS Inc) for Windows. For exploratory RCT such as these, sample sizes are not calculated based on formal power analyses. For this trial, a sample size of 200 patients was chosen, which was considered a sufficient size for a representation of the relevant variation in the target group.

## Results

### Overview

**Figure 1:**
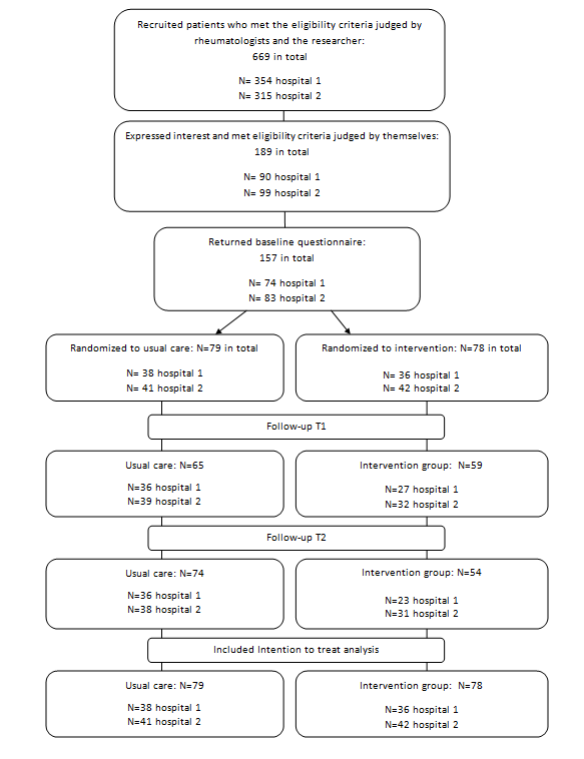
CONSORT flow diagram.

In total, 669 patients were eligible and invited. Of these, 191 patients expressed interest and 189 met the inclusion criteria (see Figure 1). In total, 157 patients completed the baseline questionnaire between January 2015 and June 2015. These patients were randomly assigned to the intervention group (n=78) and the control group (n=79), stratified by hospital. At T1, 59 in the intervention group and 65 in the control group filled in the questionnaire. At T2, 54 patients in the intervention group and 74 patients in the control group completed the questionnaire. Overall, in the intervention group, few patients (69%, 54/78) participated at T2 than in the control group (94%, 74/79). Most of these patients gave the burden of their illness as the reason for dropout. Some patients refused to fill in the questionnaire at T1 but completed the questionnaire at T2, which explains the higher number of patients who filled in the questionnaire at T2 compared with T1. Differences in demographics and disease-related characteristics between the group of patients who refused to fill in the questionnaire at T2 and the group who returned the questionnaire at T2 were small (<10%), which indicated that dropout did not influence the outcomes.

**Table 2:** Demographics and disease-related characteristics at baseline.

Characteristics		Control group		Intervention group	
		N	Statistics	N	Statistics
Age (years), mean (SD)			62.9 (10.2)		61.0 (11.3)
**Gender, %**					
	Men	27	34	27	35
	Women	52	66	51	65
Disease duration, median (25th, 75th percentiles)		79	17 (6.0, 26)	77	9 (5.0, 19.5)
**Education level, %**					
	Low	28	35	10	13
	Medium	28	35	43	55
	High	23	29	25	32
**Employment status, %**					
	Not working	50	63	41	53
	Part-time working	7	9	7	9
	Working	22	28	30	39
Physical disability (M-HAQ^a^), median (25th, 75th percentiles)		79	0.5 (0.1, 1.4)	78	0.6 (0.1, 1.1)
NRS pain today, mean (SD)		79	3.3 (2.3)	77	3.2 (2.2)
NRS mean pain last 2 weeks, mean (SD)		79	3.9 (2.3)	78	3.6 (2.3)
NRS fatigue today (NRS), mean (SD)		79	4.1 (2.5)	78	3.8 (2.4)
NRS mean fatigue last 2 weeks, mean (SD)		79	4.3 (2.4)	78	4.3 (2.3)

^a^M-HAQ: Modified Health Assessment Questionnaire.

### Baseline Characteristics of Patients

Demographics and disease-related characteristics at baseline were compared for the control group and the intervention group, as shown in Table 2. The only significant between-group difference in the patient characteristics was education level (*P*=.003). Fewer patients in the intervention group had a lower education level (12.8% vs 35.4%) and more patients had a moderate (55.1% vs 35.4%) or higher education level (32.1% vs 29.1%). Some patients who filled in a paper questionnaire did not complete all items, which explains the missing data in Tables 3 and 4.

### The Outcome Measurements at Baseline and Follow-up

Table 3 gives an overview of the mean scores of outcome measurements of the patients in the intervention and control groups at baseline and after 6 and 12 months. The baseline scores of the 2 groups did not differ significantly.

**Table 3:** Mean scores of outcome measurements on baseline, T1 and T2 of control and intervention groups.

Scales, group		Baseline (T0)		T1		T2	
		N	Mean (SD)	N	Mean (SD)	N	Mean (SD)
**PAM^a^ (10-65)**							
	Control	57	46.9 (4.9)	49	47.7 (4.8)	45	47.8 (3.8)
	Intervention	47	47.2 (3.7)	35	46.7 (6.9)	31	47.8 (2.9)
**SMAS-S^b^ (0-60)**							
	Control	79	36.0 (6.3)	75	37.9 (6.8)	74	37.6 (6.8)
	Intervention	78	36.7 (7.1)	57	39.4 (6.4)	54	38.8 (7.0)
**RASE^c^ (28-140)**							
	Control	79	99.4 (12.7)	75	101.5 (10.6)	74	99.9 (11.6)
	Intervention	78	102.9 (10.2)	57	101.9 (10.3)	54	102.0 (7.4)
**PEPPI-5^d^ (5-25)**							
	Control	79	21.6 (3.0)	75	21.0 (3.2)	73	20.6 (3.4)
	Intervention	78	21.2 (3.3)	57	21.3 (3.1)	54	20.8 (3.1)
**RAND physical functioning (0-100)**							
	Control	78	58.1 (27.0)	75	59.4 (26.5)	74	61.8 (25.9)
	Intervention	77	61.7 (26.1)	57	65.9 (27.3)	54	65.9 (26.7)
**RAND social functioning (0-100)**							
	Control	79	73.3 (2476)	75	72.7 (22.3)	74	73.1 (22.4)
	Intervention	78	71.3 (20.8)	57	77.0 (19.6)	54	70.8 (24.3)
**RAND physical role limitations (0-100)**							
	Control	79	49.1 (43.6)	75	51.11 (45.3)	74	49.0 (43.1)
	Intervention	78	49.0 (43.3)	56	57.9 (42.0)	54	49.1 (44.2)
**RAND emotional role limitations (0-100)**							
	Control	79	75.1 (40.5)	73	84.9 (35.2)	74	78.8 (39.2)
	Intervention	77	80.1 (36.4)	54	85.2 (31.5)	54	78.4 (37.3)
**RAND mental health (0-100)**							
	Control	78	54.7 (14.3)	75	72.6 (16.7)	74	76.1 (14.6)
	Intervention	78	52.6 (13.4)	56	76.5 (12.0)	54	75.9 (13.8)
**RAND vitality (0-100)**							
	Control	78	51.2 (22.7)	75	53.9 (21.6)	74	56.3 (21.2)
	Intervention	78	53.1 (19.4)	56	61.2 (15.1)	54	62.5 (14.5)
**RAND pain (0-100)**							
	Control	79	59.9 (21.3)	75	60.8 (22.2)	74	66.1 (21.8)
	Intervention	78	64.3 (22.3)	57	67.1 (21.0)	54	63.9 (22.1)
**RAND general health perception (0-100)**							
	Control	79	52.5 (18.7)	75	47.8 (18.3)	72	48.1 (17.5)
	Intervention	77	52.7 (20.8)	57	52.3 (19.5)	48	50.4 (19.1)
**RAND health change (0-100)**							
	Control	79	44.9 (21.2)	75	50.3 (19.9)	74	44.3 (19.7)
	Intervention	78	47.8 (23.2)	57	51.3 (20.8)	54	43.5 (23.9)
**NRS^e^ pain today (0-10)**							
	Control	79	3.3 (2.3)	75	3.2 (2.2)	72	3.0 (2.2)
	Intervention	77	3.2 (2.2)	57	3.0 (2.3)	48	3.3 (2.3)
**NRS mean pain last 2 weeks (0-10)**							
	Control	79	3.9 (2.3)	75	3.8 (2.1)	72	3.6 (2.2)
	Intervention	78	3.6 (2.3)	57	3.4 (2.3)	48	3.9 (2.4)
**NRS fatigue today (0-10)**							
	Control	79	4.1 (2.5)	75	3.8 (2.6)	72	3.7 (2.3)
	Intervention	78	3.8 (2.4)	57	3.4 (2.4)	48	3.6 (2.4)
**NRS mean fatigue last 2 weeks (0-10)**							
	Control	79	4.3 (2.4)	75	4.2 (2.6)	72	4.2 (2.4)
	Intervention	78	4.3 (2.3)	57	3.7 (2.1)	48	4.0 (2.2)
**MPCI-F^f^ (4-32)**							
	Control	79	14.1 (4.8)	75	14.1 (4.7)	74	13.6 (4.3)
	Intervention	78	14.1 (3.9)	57	13.3 (3.3)	54	13.6 (3.2)

^a^PAM: Patient Activation Measurement.

^b^SMAS-S: short Self-Management Ability Scale.

^c^RASE: Rheumatoid Arthritis Self-Efficacy.

^d^PEPPI-5: Perceived Efficacy in Patient-Physician Interaction.

^e^NRS: numerical rating scale.

^f^MPCI-F: Modified Pain Coping Inventory for Fatigue.

In Table 4, the estimated differences between the intervention and control groups of the intention-to-treat analysis at 6 and 12 months are presented. Overall, the scores show no significant differences and small effect sizes. Only the outcome measurement of the subscale RAND-36 vitality at T2 (5.41 95% CI 0.16-10.65, *P*=.04) showed a significant difference with respect to T0, with a small effect size (Cohen *d*) of 0.01 in favor of the intervention group. Floor and ceiling effects were explored for all specified outcomes at baseline but were not found.

### Sensitivity Analysis

#### Baseline Characteristics of Patients

High users of the intervention scored statistically significantly better than low users of the intervention on the following baseline characteristics: physical disability (M-HAQ; *P=*.03), RAND-36 subscale social functioning (*P=*.02), RAND-36 subscale physical role limitations (*P=*.03), RAND-36 pain (*P*=.03), and all the NRS scales, that is, pain today (*P*=.002), mean pain last 2 weeks (*P=*.02), fatigue today (*P*<.001) and mean fatigue last 2 weeks (*P*<.001; see Table 5).

After performing the sensitivity analysis, a statistically significant effect was found for the group with high usage on the subscale RAND-36 general health perception after 12 months (9.65, 95% CI 0.83-18.48, *P=*.03), with a small effect size of 0.02 (Table 6). No floor and ceiling effects were found for any of the specified outcomes at baseline in the groups with low or high usage.

**Table 4:** The estimated group differences between intervention and control groups after intention-to-treat analysis at 6 months and 12 months after baseline.

Scales	6 months after baseline				12 months after baseline			
	T0-T1 change	95% CI	*P* value^a^	Cohen *d*	T0-T2 change	95% CI	*P* value^a^	Cohen *d*
PAM^b^ (10-65)	–0.7	–3.4 to 1.5	.44	0.0	–0.1	–1.6 to 1.5	.93	0.00
SMAS-S^c^ (0-60)	0.3	–1.4 to 2.0	.72	0.0	0.7	–1.1 to 2.5	.43	0.03
RASE^d^ (28-140)	–2.1	–4.9 to 0.8	.16	0.0	0.3	–2.2 to 2.9	.81	0.00
PEPPI-5^e^ (5-25)	0.4	–0.5 to 1.2	.40	0.0	0.3	–0.7 to 1.3	.51	0.03
RAND^f^ physical functioning (0-100)	2.5	–3.3 to 8.1	.40	0.00	–0.2	–5.4 to 5.1	.96	0.00
RAND social functioning (0-100)	4.1	–1.5 to 9.6	.15	0.0	–2.7	–9.2 to 3.8	.42	–0.01
RAND physical role limitations (0-100)	5.6	–7.0 to 18.2	.38	0.00	–2.8	–14.9 to 9.3	.65	0.00
RAND emotional role limitations (0-100)	–3.2	–14.1 to 7.6	.56	0.00	–3.9	–16.0 to 8.3	.53	0.00
RAND mental health (0-100)	2.8	–1.1 to 6.8	.16	0.0	0.9	–3.0 to 4.7	.66	0.00
RAND vitality (0-100)	3.4	–1.5 to 8.3	.17	0.0	5.4	0.2 to 10.7	.04^g^	0.01
RAND pain (0-100)	2.6	–3.7 to 8.9	.42	0.0	–6.1	–12.5 to 0.4	.06	–0.01
RAND general health perception (0-100)	2.2	–2.2 to 6.7	.33	0.0	–0.1	–4.5 to 4.4	.98	0.00
RAND health change (0-100)	0.1	–6.8 to 7.1	.97	0.00	–1.4	–9.0 to 6.2	.72	0.00
NRS^h^ pain today (0-10)	0.0	–0.6 to 0.7	.97	0.00	0.5	–0.1 to 1.2	.13	0.10
NRS mean pain last 2 weeks (0-10)	0.0	–0.7 to 0.6	.97	0.00	0.7	0.0 to 1.4	.60	1.13
NRS fatigue today (0-10)	0.2	–0.5 to 0.8	.66	0.0	0.3	–0.4 to 0.9	.46	0.01
NRS mean fatigue last 2 weeks (0-10)	–0.23	–0.9 to 0.4	.45	–0.1	0.1	–0.6 to 0.7	.81	0.00
MPCI-F^i^ (4-32)	0.1	–0.8 to 0.9	.90	0.00	0.3	–0.7 to 1.2	.58	0.01

^a^Values represent outcomes of the ITT analysis without confounders. After adding confounders, no changes in values appear.

^b^PAM: Patient Activation Measurement.

^c^SMAS-S: short Self-Management Ability Scale.

^d^RASE: Rheumatoid Arthritis Self-Efficacy.

^e^PEPPI-5: Perceived Efficacy in Patient-Physician Interaction.

^f^RAND-36: General Health Status.

^g^Significant differences (*P*<.05) between control and intervention groups.

^h^NRS: numerical rating scale.

^i^MPCI-F: Modified Pain Coping Inventory for Fatigue.

**Table 5:** Scores at baseline for the groups with a low and high usage of the intervention: demographic characteristics, disease-related characteristics, and outcome measures.

Characteristics and outcome measures at baseline		Low usage		High usage		*P* value
		N	Statistics	N	Statistics	
Age (years), mean (SD)		29	63.8 (10.5)	40	58.9 (10.8)	.06
**Gender, %**						.55
	Men	10	33	14	35
	Women	20	67	26	65
Disease duration*,* (median (25th, 75th percentiles))		29	8,0 (4,5, 22,5)	40	8,5 (5,0, 18,7)	.80
**Education level, %**						.78
	Low	3	10	5	13
	Middle	19	63	22	55
	High	8	28	13	33
**Employment status, %**						.28
	Not working	22	73	23	58
	Working	8	27	17	43
Physical disability (Modified Health Assessment Questionnaire), (median [25th, 75th percentiles])		30	1.1 (0.2, 1.6)^a^	40	0.5 (0.1, 1,0)	.03^a^
PAM^b^ (10-65), mean (SD)		20	48.0 (3.3)	20	46.2 (3.8)	.11
SMAS-S^c^ (0-60), mean (SD)		30	36.5 (7.3)	40	37.7 (7.0)	.48
RASE^d^ (28-140), mean (SD)		30	102.1 (10.9)	40	103.4 (9.1)	.58
PEPPI-5^e^ (5-25), mean (SD)		30	21.5 (3.9)	40	21.2 (2.8)	.68
RAND^f^ physical functioning (0-100), mean (SD)		29	54.3 (28.3)	40	66.3 (24.6)	.07
RAND social functioning (0-100), mean (SD)		30	64.6 (24.8)	40	77.8 (17.1)	.02^a^
RAND physical role limitations (0-100), mean (SD)		30	36.7 (43.9)	40	60.0 (42.7)	.03^a^
RAND emotional role limitations (0-100), mean (SD)		29	74.7 (41.5)	40	85.8 (33.7)	.24
RAND mental health (0-100), mean (SD)		30	72.1 (16.1)	40	78.7 (11.6)	.06
RAND vitality (0-100), mean (SD)		30	53.1 (22.9)	40	61.7 (15.4)	.08
RAND pain (0-100), mean (SD)		30	56.9 (25.5)	40	69.8 (19.2)	.03^a^
RAND general health perception (0-100), mean (SD)		29	46.0 (19.4)	40	54.0 (17.6)	.08
RAND health change (0-100), mean (SD)		30	43.3 (20.7)	40	52.5 (24.6)	.10
NRS^g^ pain today (0-10), mean (SD)		29	4.3 (2.5)	40	2.5 (1.8)	.002^a^
NRS mean pain last 2 weeks (0-10), mean (SD)		30	4.4 (2.5)	40	3.1 (2.1)	.02^a^
NRS fatigue today (0-10), mean (SD)		30	4.8 (2.4)	40	3.0 (2.2)	<.001^a^
NRS mean fatigue last 2 weeks (0-10), mean (SD)		30	4.8 (2.4)	40	3.0 (2.2)	<.001^a^
MPCI-F^h^ (4-32), mean (SD)		30	15.0 (4.8)	40	13.2 (3.0)	.08

^a^Significant differences (*P*<.05) between the group low and high users.

^b^PAM: Patient Activation Measurement.

^c^SMAS-S: short Self-Management Ability Scale.

^d^RASE: Rheumatoid Arthritis Self-Efficacy.

^e^PEPPI-5: Perceived Efficacy in Patient-Physician Interaction.

^f^RAND-36: General Health Status.

^g^NRS pain/fatigue: Numeric Rating scales pain/fatigue.

^h^Coping with fatigue: Modified Pain Coping Inventory for Fatigue.

**Table 6:** The estimated difference between the group with low and high usage of the intervention after sensitivity analysis at 6 months and 12 months after baseline.

Scales	6 months after baseline				12 months after baseline			
	T0-T1 change	95% CI	*P* value^a^	Cohen *d*	T0-T2 change	95% CI	*P* value^a^	Cohen *d*
PAM^b^ (10-65)	2.4	–1.7 to 6.4	.24	0.12	0.0	–2.9 to 2.9	>.99	0.00
SMAS-S^c^ (0-60)	–0.4	–3.4 to 2.7	.82	0.00	1.3	–2.0 to 4.5	.44	0.02
RASE^d^ (28-140)	–1.7	–6.8 to 3.4	.52	–0.00	–0.6	–5.3 to 4.1	.81	0.00
PEPPI-5^e^ (5-25)	–1.0	–2.5 to 0.5	.20	–0.11	–0.1	–1.9 to 1.7	.93	0.00
RAND^f^ physical functioning (0-100)	9.2	–0.7 to 19.2	.07	0.01	2.2	–7.4 to 11.8	.65	0.00
RAND social functioning (0-100)	1.5	–8.4 to 11.4	.76	0.00	5.3	–6.7 to 17.4	.38	0.01
RAND physical role limitations (0-100)	7.4	–14.7 to 29.5	.51	0.00	3.7	–18.6 to 25.9	.74	0.00
RAND emotional role limitations (0-100)	16.1	–3.6 to 35.7	.11	0.01	–1.7	–24.5 to 21.0	.88	0.00
RAND mental health (0-100)	0.8	–6.3 to 7.9	.83	0.00	–4.2	–11.2 to 2.8	.24	–0.02
RAND vitality (0-100)	2.9	–5.6 to 11.5	.50	0.01	–1.2	–10.8 to 8.4	.81	0.00
RAND pain (0-100)	1.7	–4.5 to 12.9	.77	0.00	8.8	–3.0 to 20.6	.14	0.02
RAND general health perception (0-100)	2.9	–5.1 to 10.8	.48	0.01	9.7	0.8 to 18.5	.03^g^	0.02
RAND health change (0-100)	8.3	–4.0 to 20.5	.19	0.02	6.4	–7.9 to 20.6	.38	0.01
NRS^h^ pain today (0-10)	0.0	–1.2 to 1.2	>.99	0.00	–0.6	–1.9 to 0.8	.41	–0.11
NRS mean pain last 2 weeks (0-10)	–0.6	–1.8 to 0.5	.29	–0.12	0.9	–2.3 to 0.6	.24	–0.16
NRS fatigue today (0-10)	0.2	–1.0 to 1.3	.73	0.03	–0.9	–2.2 to 0.5	.22	–0.14
NRS mean fatigue last 2 weeks (0-10)	0.2	–1.0 to 1.3	.78	0.03	–0.5	–1.7 to 0.8	.51	–0.08
MPCI-F^i^ (4-32)	–0.2	–1.7 to 1.3	.76	–0.01	–0.4	–2.1 to 1.3	.67	–0.01

^a^Values represent outcomes of the ITT analysis without confounders. After adding confounders, no changes in values appear.

^b^PAM: Patient Activation Measurement.

^c^SMAS-S: short Self-Management Ability Scale.

^d^RASE: Rheumatoid Arthritis Self-Efficacy.

^e^PEPPI-5: Perceived Efficacy in Patient-Physician Interaction.

^f^RAND-36: General Health Status.

^g^Significant differences (*P*<.05) between control and intervention groups.

^h^NRS: numerical rating scale.

^i^MPCI-F: Modified Pain Coping Inventory for Fatigue.

## Discussion

### Principal Findings

This study aimed to evaluate the efficacy of a Web-based self-management enhancing program in patients with RA in an explorative trial on 6 outcomes: self-management behavior, self-efficacy, general health status, coping with fatigue, and the level of pain and fatigue. Results show no remarkable statistically significant difference between the intervention and control group. Moreover, effect sizes were low. Consequently, the results of this exploratory show no convincing trend regarding the efficacy of the program. This was unexpected as the theory-based intervention was carefully designed, according the IM steps, on the basis of patients support needs [37,38]. In addition, the range of outcome measures were selected carefully, and the study was well-performed. Randomization was successful, and the number of missing was limited. It was thought that the size was adequate for a pilot study (N=157).

Notably, the lack of a trend for a positive result is not in line with other studies, showing that self-management programs seem to be promising for patients with a chronic illness, including arthritis [15,35]. However, these studies cannot be compared with each other in a straightforward manner because of the various self-management approaches (eg, offering weekly vs nonweekly Web-based courses, with face-to-face help or without), various contents of the self-management programs, and the different outcome measures used in these studies [15,39]. For example, it is unexpected that our Web-based program yielded no results for RA patients, whereas the Web-based program evaluated by Lorig and colleagues [14] concluded that RA patients showed increased self-efficacy and improved health status for 4 of the 6 health status measures that were included [14]. These different results may be explained by the different questionnaires used for the same outcomes, that is, self-efficacy and health status.

Moreover, differences in the content and delivery of the programs could be a reason for the different results. Other programs focused on different topics (eg, pain/stress management, problem solving and nutrition, which were not covered by our program). In our program, patients received no help with logging into the program or using the program in contrast to the program described by Lorig et al [14], where patients received help and were encouraged to use the program. Patients could choose which modules to work through and follow it at their own speed. In the program described by Lorig et al [14], peer moderators helped patients log in and encouraged them to use the weekly program and moderate posts that patients could leave on the program website [14].

There are potentially 5 reasons for the lack of efficacy of our Web-based program: (1) the use of inappropriate outcome measures, (2) individual patients had no need for self-management support, (3) low usage of the program/high dropout of the intervention group, (4) inadequate embedding of the program in health care, and (5) not selecting the appropriate patients.

First, in the case of inappropriate outcome measures, it could be that the carefully selected validated questionnaires still did not exactly measure the pursued behavior changes formulated in the performance objectives. That is, the intervention aimed to result in specific self-management behaviors. The validated questionnaires comprised more generic questions and therefore did not exactly measure these specifically formulated behavioral changes in performance objectives (Table 1). However, it was expected that a positive significant result would be found on the RASE questionnaire, as this measures task-specific self-efficacy for patients with RA with items closely related to the specific formulated performance objectives. Finding no positive results suggests that it is possible that our intervention did not support patients in increasing their level of self-efficacy. This could mean that the absence of positive results is less driven by the choice of outcomes than by the other points discussed below.

Second, it could be that recruited patients did not have a perceived need for enhancing self-efficacy when they agreed to participate in the program. Although the program was developed on the basis of the support needs for self-management of RA patients, individual participating patients in this study were not asked whether, and if yes, what kind of support needs they had for self-management. It could be that patients differ in their needs and more tailoring toward individuals is needed, for example, preselection of the offered modules.

Third, the low usage of the program by patients in the intervention group could have resulted in finding only a significant effect on RAND-36 vitality, with a small effect size. The low usage of the program can have several reasons. As stated above, patients could have not felt a need for support. Another reason could be that patients were not motivated to change their behavior or had a negative attitude toward the Web-based program. The program comprised several elements to stimulate patients’ usage of the program, such as persuasive texts or modeling videos. It could be that these elements did not work or that elements were lacking in the program. Moreover, the characteristics of the Web-based program, for example, attractiveness or the ease of logging in, are factors that could have influenced patient usage of the program. It was also notable that patients in the intervention group dropped out more than patients in the control group. A high dropout rate is a common finding in Web-based programs [40,41]. Crutzen et al (2015), gave as possible explanation for these higher dropout rates that patients in an intervention group have several expectations of the intervention. In cases where these expectations are not met or if patients feel the intervention is not supporting them, patients will refuse to fill in the measurements and will not revisit the program [42]. In this study, patients in the intervention group were significantly higher educated than in the control group. It could be that higher educated patients use more resources that could support them (eg, support of health professionals), which could lead to lower usage of the program.

Fourth, this program was not adequately embedded in patient care. Although nurses brought the program to the attention of intervention group patients during their consultation, they did not discuss the self-management topics of the program with patients to continue the support for self-management during consultations. It has been shown that self-management programs with the possibility of interacting with health professionals (blended care) can lead to positive results [14,43].

Fifth, it could be that there was a selection bias in this study. Rheumatologists selected patients with diagnosis RA, aged 18 years, or were invited by letter to participate in this study, in collaboration with rheumatologists until the required number of 190 patients were reached. Probably, rheumatologists mainly selected the patients who had a low functional disability (health assessment questionnaire) as in their opinion, these patients would benefit of a self-management program the most.

### Recommendations

Given the results of this study, relevant recommendations for future studies and practice can be given. First, using a questionnaire with questions referring to the program objectives is recommended to measure the effects in patient behavioral change [38]. For example, one of the performance objectives of this Web-based program, *“* *set boundaries in their work situation,”* could be evaluated with an item like *“* *I’m able to set boundaries with my colleagues in my work situation”* (measuring skills). Patients can set their own objectives in the program, using goal setting as a strategy. Goal setting requires that patients set a clear, specific, and achievable goal to change their behavior. This concrete formulation of the goal ensures that the behavioral change is measurable [44].

Second, before inclusion, it is recommended to investigate whether patients have a need for self-management support and if so, what kind of support they need. A next step is to decide if patient support needs are handled in the program and to tailor the program to their support needs. This can avoid patients feeling that the program did not support them, which often results in no revisits. Investigation of support needs could take place over the telephone. This also offers the possibility of helping patients formulate their support needs, which is difficult to do in general.Third, to increase the usage of the program and limit dropout, during the development phase, it is important to pay attention to factors that could enhance usage of the program (first visit, staying on the website, revisits).Patients input, in combination with attention to dissemination, reach, adoption and implementation (emphasized in diffusion theory or RE-AIM theory), could be used to identify factors [45,46]. Moreover, qualitative research to explore the reasons for low usage should be conducted.

Fourth, to embed the program in regular health care, it is important that patient needs are also recognized by their rheumatologists or specialized rheumatology nurse and be used as a starting point during consultation. Nurses could also assist patients in performing exercises mentioned in the program, reminding patients to log on to the website and encourage patients to maintain their self-management behavior. Fifth, to increase the usage and efficacy of the program, a specific patient selection is needed. Further research is needed to assess which patient characteristics influence the use of a Web-based program and the outcomes, for example, by performing subgroup analysis among groups with a low or high functional disability or by assessing their level of motivation to use the program. This can determine which inclusion criteria should be used to select patients likely to benefit most.

### Conclusions

In conclusion, although there is external evidence in favor of the efficacy of Web-based self-management interventions [14,15], it is not recommended to conduct a larger trial yet. As advised by the MRC framework, a detailed process evaluation of the program should be conducted to gain thorough insight into the implementation of the program, the working elements of the program and the usage of the program by patients, which could be both important conditions for the success of a self-management program. This could also satisfy the need for attention to the usage and the perceived impact of the program to find out for whom this sort of program will work [47].

## Abbreviations

IM: intervention mapping
M-HAQ: Modified Health Assessment Questionnaire
MPCI-F: Modified Pain Coping Inventory for Fatigue
MRC: Medical Research Council
NRS: Numeric Rating Scale
PAM-13: Patient Activation Measurement
RA: rheumatoid arthritis
RASE: Rheumatoid Arthritis task-specific Self-Efficacy Questionnaire
RCT: randomized controlled trial
SMAS-S: Short Self-Management Ability Scale

## Appendix

Multimedia Appendix 1 CONSORT-eHEALTH checklist (V 1.6.1).
